# Improving access to melarsomine therapy: treating canine heartworm infection in a high-volume, outpatient community clinic setting

**DOI:** 10.1186/s13071-024-06153-4

**Published:** 2024-03-08

**Authors:** Meghan B. Still, Dana Tedesco, Christina Hawkins, Holly Putnam

**Affiliations:** 1 Research, Data, and Analytics, Emancipet, Austin, TX USA; 2 Clinical Services Group, Emancipet, Austin, TX USA; 3 Community Medicine, American Society for the Prevention of Cruelty to Animals, New York, NY USA

**Keywords:** Heartworm, *Dirofilaria immitis*, Arsenical adulticide treatment, Melarsomine, Accessible veterinary care, Retention rate, Adverse reactions, Therapy adherence

## Abstract

**Background:**

Models that provide high-quality veterinary care for more affordable prices are emerging, but not well documented outside of wellness and preventative care. Effective treatment guidelines for heartworm disease have been developed by the American Heartworm Society; however, not all owners are able to access treatment due to the high costs associated with sick and emergency care services.

**Methods:**

To increase access to high-quality adulticidal treatment of canine heartworm disease, we developed and implemented a technician-leveraged heartworm treatment protocol for high-volume, outpatient community clinic settings based on the American Heartworm Society guidelines. Modifications were few and included limited pre-treatment blood work, pre-injection sedation, post-injection pain medication, and a reduced exercise restriction period. We monitored retention rates for 556 dogs throughout treatment, evaluated treatment success (defined as no antigen detection 9 months post treatment) for patients that returned for post-treatment antigen testing, and reported on adverse reactions and therapy adherence throughout treatment.

**Results:**

Of the patients that began adulticide therapy, 539/556 (97%) successfully completed the three-injection series. No microfilariae were detected in 99% (428/433) of those who returned for post-injection microfilaria testing. Among those that returned for or reported the results of post-injection antigen testing, no antigen was detected for 99% (245/248) and no microfilariae were detected for 99.5% (200/201). During the course of treatment, 483/539 (90%) of patients experienced at least one adverse reaction, with the most frequently reported types being behavioral and injection site reactions. 25/539 (4.6%) of owners sought additional medical care for adverse reactions at some point during the treatment course. The overall mortality rate was 1.3% (7/556).

**Conclusions:**

This study represents the first evaluation of a heartworm treatment protocol optimized for implementation in a high-volume, outpatient community clinic setting. Our findings align with those previously reported in private practice or tertiary referral centers, illustrating that through the inclusion of pre-treatment blood work, employing short-acting or reversible sedatives, ensuring proper analgesia, minimizing the use of ancillary diagnostics, reducing the duration of in-clinic monitoring while focusing on outpatient care, and maximizing technician involvement, we can deliver effective and safe melarsomine therapy at a more affordable cost to pet owners.

**Graphical Abstract:**

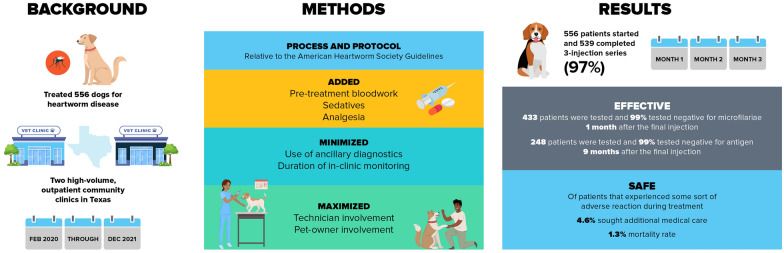

## Background

Canine heartworm disease, caused by the filarial nematode, *Dirofilaria immitis*, is a serious threat to the health and welfare of dogs across the United States, with more than 100,000 new cases reported each year [1]. While reported annual heartworm disease incidence for the United States is between 1 and 2% [2, 3], mean prevalence can be much higher for populations at high-risk of infection (i.e., unowned dogs living where favorable climatic conditions and infection reservoirs coincide). For example, in southeastern Florida where competent mosquito vectors are present year-round, up to 28% of unowned dogs admitted to shelters are heartworm-positive [4]. Despite widespread availability of heartworm preventive medications, incidence continues to increase, particularly in the southeastern United States, as a result of expanding mosquito vector range associated with climate change, canine host movement from heartworm endemic regions and increased testing and recognition of the disease [3, 5].

The American Heartworm Society (AHS) has developed treatment guidelines for heartworm disease that consists of 90 days of therapy and subsequent follow-up testing [5]. These guidelines have become the standard recommended treatment for heartworm disease within the United States. Despite the general availability, not all pet owners can access the veterinary care required to treat heartworm disease in their pets. A recent report from the Access to Veterinary Care Coalition found that, among the multitude of barriers preventing pet owners from accessing veterinary care, the most frequently reported barrier is financial [6]. The report further revealed that half of the surveyed respondents who faced challenges in accessing preventive care for their pets expressed a desire for heartworm, flea, or tick preventives but were unable to obtain them. The AHS treatment protocol for heartworm disease may also be cost-prohibitive for many owners. In fact, one study found that when owners were able to access heartworm treatment, 50% of heartworm treatment plans were modified from the recommended plan due to the pet owner’s financial concerns [7].

Low-cost, high-volume veterinary clinics aim to reduce the financial barriers associated with accessing veterinary care. Effective low-cost, high-volume models exist for many aspects of veterinary care including vaccinations, wellness visits, and spay/neuter surgeries [8–10]. While a brief internet search can yield multiple low-cost heartworm treatment options, no studies to date have evaluated a low-cost, high-volume approach to heartworm treatment.

For this study, we evaluated a heartworm treatment program based on AHS guidelines and modified for low-cost, high-volume community clinics. To minimize costs passed on to the owners, the operational model of these clinics is highly technician-dependent and based on high-volume, outpatient care. Thus, our primary goal was to determine whether an operational model for low-cost, high-volume, outpatient treatment of heartworm disease is as effective and safe as AHS protocol models previously reported in private practice or tertiary referral centers.

Here, we provide insights into the retention rate at each stage of heartworm treatment. We then compare the treatment success, defined as no antigen detected (NAD) 9 months post-treatment, with those previously reported in private practice or tertiary referral centers. Additionally, we report clinical signs observed by veterinary technicians (VTs) or veterinary assistants (VAs) shortly after each melarsomine injection prior to discharging the patient for outpatient management, along with the associated adverse reactions reported by owners within 10 days of discharge. Finally, we delve into adherence with medication and exercise restriction prescribed during treatment.

## Methods

### Study locations and patient inclusion

The study was conducted at two high-volume, low-cost community clinics within the same nonprofit organization, both centered in low-income, urban communities in Texas. All data were collected between February 2020 and December 2021. Canine patients were included in the study if they met each of the following criteria: (1) The patient had been previously diagnosed as heartworm-positive, either at one of the study clinics or another veterinary facility. (2) The patient visited one of the two study clinics for an initial heartworm disease evaluation between February and September of 2020, and the owner or foster caregiver provided informed consent to treatment and to use of their pet's medical data for purposes such as to benefit the care of other pets. (3) The patient had a second positive test using one of two methods at the time of the initial evaluation: (i) microscopic examination of a direct blood smear with microfilariae present or (ii) positive antigen test with either an IDEXX SNAP Heartworm RT Test (SNAP) or a Zoetis WITNESS Heartworm Rapid Test (WITNESS). (4) The patient was diagnosed with class I (mild), class II (moderate), or class III (severe) heartworm disease based on clinical signs outlined in the AHS guidelines for staging heartworm disease severity [5]. (5) The patient was prescribed melarsomine therapy based on AHS guidelines. Patients were not excluded based on demographics; therefore, the study sample includes all sexes, ages, breeds, and ownership statuses (whether owned or fostered at the beginning of treatment) that met the inclusion criteria outlined above.

### Treatment protocol

Except where modifications are noted, the treatment protocol followed the 2014 AHS guidelines for heartworm treatment dosing and schedule (Table 1) [5]. Modifications to the 2014 AHS guidelines were few and included the following:

- Pre-treatment blood work: A blood chemistry panel was performed using a portable chemistry analyzer (VETSCAN VS2 Chemistry Analyzer, Abaxis, Union City, CA, USA) prior to the first melarsomine injection. Blood urea nitrogen (BUN), creatinine, alkaline phosphatase (ALKP), alanine aminotransferase (ALT), total protein, and blood glucose were measured.
- Pre-injection sedation: Prior to each melarsomine injection, all patients were administered trazodone 5–10 mg/kg orally (PO). Patients continuing to show signs of high fear, anxiety, and stress (FAS) following trazodone administration were given a secondary sedative. These patients received an intramuscular injection (IM) of butorphanol 0.2 mg/kg and dexmedetomidine 0.01 mg/kg at a site distant from the standard melarsomine injection sites. A minimum of 30 min was allowed between oral or IM sedative administration and melarsomine injection.
- Post-injection pain medication: Following the first and second melarsomine injections, all patients were started on tramadol 5 mg/kg PO twice daily (BID) × 3 days upon their return home.
- Reduced exercise restriction period: Owners were advised to restrict their pet’s activity for 30 days following each melarsomine injection, or for 60 days total throughout treatment. Restricted exercise was described to owners as kennel confinement except for feeding and leash walking for bathroom purposes only. Owners were advised to return to normal patient activity after the 60 days of restricted exercise was completed.

**Table 1:** Heartworm treatment schedule

Day	Treatment
Day 0: Initial evaluation appointment	• Confirmation heartworm test • Doxycycline 10 mg/kg BID × 30 days, monthly macrocyclic lactone heartworm preventive • Trazodone 5–10 mg/kg PO dispensed to administer prior to each melarsomine injection^a^
Day 60: First melarsomine injection appointment	• Blood chemistry ﻿panel^a^ • Melarsomine 2.5 mg/kg IM • Prednisone 0.5 mg/kg BID × 7 days, once daily (SID) × 7 days, then every 48 h × 14 days • Tramadol 5 mg/kg PO BID × 3 days^a^ • Continue monthly macrocyclic lactone heartworm preventive • Exercise restriction for 30 days^a^
Day 90: Second melarsomine injection appointment	• Melarsomine 2.5 mg/kg IM • Prednisone 0.5 mg/kg BID × 7 days, SID × 7 days, then every 48 h × 14 days • Tramadol 5 mg/kg PO BID × 3 days^a^ • Continue monthly macrocyclic lactone heartworm preventive • Exercise restriction for 30 days^a^
Day 91: Third melarsomine injection appointment	• Melarsomine 2.5 mg/kg IM
Day 120: Post-injection microfilaria test appointment	• Direct blood smear to evaluate for presence of microfilariae
Day 365: Post-injection antigen test appointment	• WITNESS test and screen for microfilariae

^a^Modifications to the American Heartworm Society guidelines [5]

#### Administration of melarsomine injections

Melarsomine injections were administered by licensed VTs and trained VAs under the direct supervision (as defined by the Texas Veterinary Licensing Act [11]) of a veterinarian (DVM) following AHS guidelines for dosing and injection technique [5]. Up to 4 ml was administered per injection site; if larger volumes were required, they were divided into two injections given in the same epaxial muscle 1 inch apart. Patients were kept in the clinic for an observation period of 2.5–4.5 h after each injection.

### Treatment process

#### Role of VT/VA and DVM

All patients were examined by a DVM at the initial evaluation and prior to each melarsomine injection. The DVM reviewed all medical histories and blood work, classified the patient’s heartworm disease based on AHS guidelines [5], prescribed appropriate treatment plans, and made all medical decisions for each patient. At each step in the treatment protocol, teams of two or three specially trained VT/VA, working under the direct supervision of a DVM, were responsible for patient check-in, obtaining medical histories, and additional information relevant to treatment, performing diagnostics, administering melarsomine injections, dispensing medications prescribed by the DVM, and monitoring and discharging patients. Standardized forms were used at each step of the treatment protocol to ensure consistent and streamlined collection of information from owners and recording of patient data.

Both licensed VTs and unlicensed VAs with previous high-volume clinical experience were trained in each step of the treatment protocol. After training, licensed VTs and unlicensed VAs were able to demonstrate equal levels of proficiency in each step of the protocol, allowing for flexibility in staff scheduling.

#### Initial patient evaluation

At the initial heartworm disease evaluation, VT/VA gathered information from owners about the patient’s current and previous medical history. The VT/VA performed confirmation microfilaria and heartworm antigen tests. If the patient was microfilariae negative, the confirmation antigen test performed was from a different manufacturer than the test used for the initial diagnosis (e.g., if a SNAP test was used in the initial diagnosis, a WITNESS test was used to confirm the initial test results).

If the patient met the inclusion criteria outlined above, the DVM prescribed a three-injection melarsomine-based heartworm treatment plan and pre-melarsomine medications were dispensed (Table 1). In rare cases, patients who otherwise fit inclusion criteria were determined to be poor candidates for melarsomine-based heartworm treatment based on the following criteria: (1) The DVM determined the patient was not a good medical candidate for melarsomine injections due to extremely poor physical condition. (2) The patient was not a good candidate for rest because they were extremely active and exercise restriction was not possible for the owner. (3) The patient was so aggressive that they were unmanageable with our handling methods and a danger to clinic staff. In these cases, the owner was referred to a full-service veterinary clinic or advised to begin the patient on non-arsenical heartworm treatment.

#### Melarsomine injection visits

For each melarsomine injection visit, information about patient history, recent adverse reactions, and adherence with the prescribed treatment plan was collected from the client by VT/VA. If the owner had not administered trazodone prior to the visit, trazodone (and additional sedation if needed as outlined above) was administered at this time by VT/VA. If this was the first melarsomine injection, pre-injection blood work was performed and reviewed by the DVM. The patient was examined by the DVM and melarsomine injections were subsequently administered by VT/VA. Patients were kept in the clinic for an observation period of 2.5–4.5 h after each melarsomine injection before being released to owners, along with take-home medications (prednisone and tramadol) and instructions for exercise restriction. Owners were asked to administer the first dose of prednisone and tramadol and start or continue exercise restriction as directed upon their return home.

Due to the limited capacity of the study sites to treat medically complex diseases, patients with pre-injection blood work suggestive of serious comorbidities were either advised to defer heartworm treatment until their condition could be stabilized at a full-service veterinary clinic or were prescribed a non-arsenical heartworm treatment. Melarsomine injections were postponed, and patients were referred to a full-service veterinary clinic if the following abnormalities were detected on blood work: (1) BUN, creatinine, ALT, or blood glucose were greater than twice the upper reference value; (2) ALKP was greater than three times the upper reference value; or (3) any elevations of the measured values in conjunction with owner-reported clinical signs suggestive of possible renal disease, liver disease, diabetes mellitus, or other serious comorbidities.

#### Post-treatment testing

VT/VA performed all post-treatment testing, which was reviewed by the DVM. A direct microscopic examination of the anticoagulated whole blood to detect microfilariae was performed 1 month and 9 months after the third melarsomine injection. A WITNESS test was performed 9 months after the third melarsomine injection. Treatment success was defined as NAD at post-treatment antigen testing 9 months after the third melarsomine injection. If the patient was microfilariae positive when tested 30 days or 9 months after the third melarsomine injection, the patient was treated with a microfilaricide and advised to retest in 4 weeks. If the patient was antigen positive 9 months after the third melarsomine injection, the patient was retreated with doxycycline followed 30 days later by two doses of melarsomine given 24 h apart, per AHS guidelines, and advised to retest in 6 months [5]. Cases of subsequent treatment and post-injection testing were not tracked and reported as a part of this study.

### Adverse reactions

#### Observations of adverse reactions

To measure adverse reactions of melarsomine, we followed Maxwell et al. [12] except that we adapted the list of adverse reactions to accommodate two forms of observation: (1) observations made by the VT/VA within the first 2.5–4.5 h of each melarsomine injection, and (2) observations made by the owner within 10 days of leaving the clinic after each melarsomine injection. After each melarsomine injection, trained VT/VA evaluated patients for the adverse reactions listed in Table 2 and recorded their observations shortly before discharging the patient to their owner. Owners were directed to monitor their pets for adverse reactions for the 10 days following the first and third melarsomine injections. At the subsequent appointment, owners were asked to complete a self-administered questionnaire about the adverse reactions observed (Table 2) as outlined in their heartworm treatment information packet. Owners were also asked about whether medical attention was sought for observed adverse reactions following the first and third melarsomine injection.

**Table 2:** Adverse reactions measured throughout treatment

Adverse reactions observed in the clinic by VT/VA following a melarsomine injection	
Behavioral	Salivation, panting, trembling, whining, reluctance to move
Local injection site	Pain, injection site swelling
Respiratory	Onset in labored breathing/dyspnea
Gastrointestinal	Vomiting, diarrhea
Immediate hypersensitivity	Pale mucous membranes, hives, facial swelling, acute collapse
Adverse reactions observed by the owner up to 10 days following a melarsomine injection	
Behavioral	Panting, trembling, whining, lethargy, reluctance to move
Local injection site	Pain, injection site swelling, redness, abscess
Respiratory	Abnormally rapid breathing, onset in labored breathing, coughing, or coughing up blood
Gastrointestinal	Vomiting, diarrhea, decreased appetite

Modified from Maxwell et al. [12]. Adverse reactions were recorded as presence/absence

#### Patient rechecks and referrals

If an adverse reaction was observed by the VT/VA in the clinic, a DVM was immediately notified. The DVM evaluated the patient and developed a treatment plan, if applicable. In the event of after-hours concerns or emergencies, owners could access a VT/VA via a dedicated phone number. For non-urgent adverse reactions, owners were advised to return to the clinic for a recheck visit the following morning. If a patient experienced an urgent adverse reaction during business hours, the owner was directed to bring the patient back to the clinic for evaluation. During all recheck visits, a DVM performed an examination and developed a treatment plan, if applicable. In the case of urgent overnight adverse reactions, a DVM was immediately notified and took over communication with the owner. If the DVM determined that the reported adverse reactions needed immediate intervention, the owner was directed to take the patient to a local veterinary emergency center for evaluation and treatment.

#### Patient mortality

Patient death, date, and circumstance were voluntarily self-reported by the pet owner.

### Therapy adherence

To measure therapy adherence, we expanded upon Maxwell et al. [12] and included all medications prescribed as a part of treatment. At each appointment, owners were asked to complete a self-administered questionnaire about adherence with medication administration and exercise restriction.

### Statistical analyses

Statistical analyses were conducted in R version R 4.1.0 GUI 1.76 High Sierra build [13] using a significance threshold of *ɑ* = 0.05. Descriptive statistics were calculated for patient retention, patient demographics, cost of treatment, treatment success, adverse reactions, and therapy adherence. Descriptive statistics are reported as percentage (total number of observations [*n*]). Continuous variables with non-normal distribution were reported as median (interquartile range [IQR]). Pearson’s Chi-square tests with Yates’ continuity corrections were used to test for differences in patient retention between patients that were fostered and patients that were owned at the beginning of treatment [13]. To account for repeated measures of the same patient across appointments, mixed models were used for all analyses using a unique identifier for each patient as a random effect. To test for differences between patients that experienced adverse reactions at the clinic and those that did not, we used a generalized linear mixed model (GLMM) with a binomial error distribution (lme4 package) [14]. All VT/VA-observed adverse reaction models were initially modeled with injection appointment (first, second, third), relative timing of melarsomine injection (defined as the deviation from the average injection time for each appointment type), timing of initial sedation (prior to or after arrival at the clinic), secondary sedation administration (presence/absence), and patient movement during administration (presence/absence), and all logical combinations as fixed effects and individual identification of patients as the random subject effect. All owner-observed adverse reaction models were initially modeled with injection appointment (first, second, third), compliance with administration of medication (doxycycline, prednisone, tramadol), and compliance with activity restriction, and all logical combinations as fixed effects and individual identification of patients as the random subject effect. All models were then subsequently reduced using an iterative model selection procedure guided by the Akaike information criterion (AIC). We used the Šidák procedure to conduct pairwise post hoc tests among categorical variables (with a significance threshold of *ɑ* < 0.05) (emmeans package) [15]. The parameter estimate and standard error (β ± SE) are reported for continuous variables. High compliance with the treatment protocol and low variation in outcomes and owner-observed adverse reactions did not permit additional statistical modeling.

## Results

### Cost of treatment

The cost of heartworm treatment varied between $225–614 per patient, depending on the size of the animal, whether the owner needed to purchase heartworm preventives, and, in a few instances, whether an additional blood chemistry was needed before proceeding with melarsomine injections. The amount actually paid by the owner varied, however, as no owner was turned away due to financial limitations.

### Sample size and retention rate

A total of 626 heartworm-positive cases visited the two study clinics during the study period. 621/626 (99%) were classified as good candidates for melarsomine-based heartworm treatment. Of the five patients that were not good candidates for melarsomine-based heartworm treatment, three exhibited extremely poor physical condition and two were not good candidates for exercise restriction. An additional 65/621 (11%) patients did not begin heartworm treatment within the 10-month data collection period. Most owners (45/65; 69%) did not cite the reason for not starting treatment. Of those who did, nine patients were switched to a non-arsenical heartworm treatment or the owner was referred to a full-service DVM, five owners were not compliant with doxycycline therapy and were asked to return upon completion, four patients died before treatment began, and two patients were re-homed or lost.

539/556 (97%) of patients that began melarsomine-based treatment completed the three-injection series (Table 3). Of the 17 patients that did not complete treatment, four patients switched to a non-arsenical heartworm treatment or the owner was referred to a full-service DVM, two patients were re-homed or the owner relocated, and one patient died (see section “Mortality Rate” for more information). The remaining 10/17 (59%) owners did not provide a reason for not completing treatment. There was no significant relationship between ownership status and retention for post-injection microfilaria (*χ*^2^ = 2.958, *df *= 1, *P* > 0.05) or antigen (*χ*^2^ = 1.769, *df *= 1, *P* > 0.05) testing. Thus, the resulting sample size for therapy completion and inclusion in subsequent analyses was 539 patients.

**Table 3:** Retention rates across each stage of the treatment plan

		Relative to previous appointment type		Cumulative		
Appointment type	Total dogs	Difference (number)	Retention rate (%)	Difference (number)		Retention rate (%)
Injection 1	556	–	–	–		–
Injection 2	541	15	97	15		97
Injection 3	539	2	99.6	17		97
Post-injection microfilaria test	442	97	82	114		80
Post-injection antigen test	253	189	57	303		46

### Patient demographics

Among the patients who completed treatment, 495/539 (92%) had class I heartworm disease, 43/539 (8.0%) had class II, and 1/539 (0.2%) had class III. The sample population consisted of 292/539 (54%) males, 427/539 (79%) under the age of seven, and was divided by size into 157/539 (29%) small (≤ 9.1 kg), 107/539 (20%) medium (≤ 22.7 kg), 260/539 (48%) large (≤ 45.4 kg), and 15/539 (2.8%) extra-large (> 45.4 kg). 454/539 (84%) of these patients were owned rather than fostered at the beginning of treatment.

### Treatment success

Of the 539 patients that completed the study therapy, 433 (80%) returned for 1-month post-injection microfilaria testing and 99% (428/433) tested negative for microfilariae. All five owners whose pets tested positive for microfilariae reported consistently giving heartworm preventives, with no lapses in therapy. Of these, two of the five microfilariae-positive patients returned to the clinic for post-injection antigen testing and microfilaria screening. Both patients were then NAD and tested negative for microfilariae. At post-injection antigen testing, one of these owners reported occasional lapses in heartworm preventive administration, while the owner reported uninterrupted therapy adherence. One of the five microfilariae-positive patients received post-injection antigen testing at another veterinary clinic and was reported to be NAD. No additional information was provided regarding the administration of heartworm preventives following the 1-month post-injection microfilaria test. Of the remaining two microfilariae positive patients, neither returned for post-injection antigen testing nor did their owner report having their pets tested at another veterinary clinic.

248/539 (46%) patients that completed the study therapy returned for 9-month post-injection antigen testing or reported antigen test results performed elsewhere; 99% (245/248) of these patients were NAD and 99.5% (200/201) tested negative for microfilariae. All three owners whose pets tested positive for antigen reported consistent administration of heartworm preventives with no lapses in therapy when asked at post-antigen testing. However, two of these owners had previously reported occasional lapses in preventive administration at some point during therapy.

### Adverse reactions and mortality rate

#### Adverse reactions

Based on VT/VA observations at the clinic, 483/539 (90%) of the patients that completed the study therapy experienced an adverse reaction at some point over the course of the three-injection series. Adverse behavioral reactions were the most common (465/539; 86%), followed by local injection site reactions (177/539; 33%) (Tables 2, 4).

**Table 4:** Adverse reactions observed at the clinic and within 10 days of a melarsomine injection

Adverse effect	Appointment type	Number of dogs	Total Dogs	Percentage (%)
VT/VA-observed adverse reactions at the clinic				
All adverse reactions		483	539	90
	Injection 1	366	479	76
	Injection 2	354	518	68
	Injection 3	288	468	62
Behavioral		465	539	86
	Injection 1	340	478	71
	Injection 2	342	518	63
	Injection 3	243	468	52
Local injection site		177	539	33
	Injection 1	80	477	17
	Injection 2	67	515	13
	Injection 3	92	468	20
Respiratory		3	539	0.6
	Injection 1	1	477	0.2
	Injection 2	2	515	0.4
	Injection 3	1	468	0.2
Gastrointestinal		16	539	3.0
	Injection 1	3	477	0.6
	Injection 2	7	515	1.4
	Injection 3	6	468	1.3
Immediate hypersensitivity		1	539	0.2
	Injection 1	0	477	0
	Injection 2	1	515	0.2
	Injection 3	0	468	0
Owner-observed adverse reactions 10 days following a melarsomine injection				
All adverse reactions		314	539	58
	Injection 1	244	505	48
	Injections 2/3	186	389	48
Behavioral		166	539	31
	Injection 1	125	467	27
	Injections 2/3	81	349	23
Local injection site		215	539	40
	Injection 1	156	502	31
	Injections 2/3	130	367	35
Respiratory		104	539	19
	Injection 1	78	503	16
	Injections 2/3	55	388	14
Gastrointestinal		136	539	25
	Injection 1	93	467	20
	Injections 2/3	73	349	21

Samples sizes vary based on missing data points within each case

The final model of VT/VA-observed adverse reactions included a two-way interaction between the relative timing of melarsomine injection and the timing of initial sedation, injection appointment, secondary sedation administration, and patient movement during administration as additional fixed effects and individual identification of patients as the random subject effect. The presence of adverse reactions significantly varied across injection appointments (GLMM: all, *P* < 0.05; Table 5). The odds of a VT/VA-observed adverse reaction were highest at the first injection with a 1.6 time decrease at the second injection (*P* = 0.025) and a 2.1 time decrease at the third injection (*P* = 0.001). In addition, the presence of VT/VA-observed adverse reactions varied significantly across the timing of sedative administration and melarsomine injections (GLMM: timing of sedative administration * relative timing of melarsomine injection, *Z* = 2.451, *P* = 0.0142; Table 5). When the patient was administered trazodone prior to arriving at the clinic, the odds of observing an adverse reaction increased (*β* = 0.005 ± 0.002) as the injection time occurred later in the day. When the patient was administered trazodone after arriving at the clinic, the odds of observing an adverse reaction decreased (*β* = −0.003 ± 0.002) as the injection time occurred later in the day.

**Table 5:** GLMM investigating the effects on VT/VA-observed adverse reactions

Fixed effect	Estimate	SE	z value	*P* ( >|z|)
(Intercept)	1.472	0.186	7.925	< 0.001*
Injection appointment 2	−0.463	0.175	−2.64	0.008*
Injection appointment 3	−0.755	0.183	−4.121	< 0.001*
Relative timing of melarsomine injection	0.005	0.002	2.245	0.0248*
Timing of sedative administration	−0.347	0.208	−1.665	0.096
Secondary sedation administration	0.123	0.229	0.0.538	0.590
Patient movement during administration	0.144	0.169	0.854	0.393
Relative timing of melarsomine injection * Timing of sedative administration	−0.008	0.003	−2.451	0.0142*

*Statistically significant

Based on owner reported observations from home, 314/539 (58%) of patients that completed the study therapy experienced an adverse reaction within 10 days of receiving a melarsomine injection. Local injection site adverse reactions were the most common (215/539; 40%), followed by behavioral (166/539; 31%), gastrointestinal (136/539; 25%), and respiratory reactions (104/539; 19%) (Tables 2, 4). 63/539 (12%) owners called with questions regarding the adverse reactions that they observed, and 25/539 (4.6%) owners sought medical attention at a full-service veterinary clinic (14/25; 56%), one of the study locations (9/25; 36%), or at an emergency clinic (2/25; 8%). No owners reported major adverse reactions within 24 h following a melarsomine injection. At post-treatment antigen testing, 11/248 (4.4%) of owners reported that the patient had residual respiratory signs.

The final model of owner-observed adverse effects included injection appointment, compliance with administration of medication (doxycycline, prednisone, tramadol), and compliance with activity restriction as fixed effects and individual identification of patients as the random subject effect. There were no significant predictors of the presence of adverse reactions (GLMM: all, *P* > 0.05).

#### Mortality rate

The mortality rate was 1.3% (7/556) across all stages of therapy. Two patients that began melarsomine therapy died after the first melarsomine injection. One death occurred 2 days after the first melarsomine injection, but no additional information was provided. The date of death was missing for one patient who did not return after the first melarsomine injection and no additional information was available. Five patients that began melarsomine therapy died after the second/third melarsomine injections. Of these patients, one owner reported a major adverse reaction within 10 days of a melarsomine injection and sought additional medical attention. That patient died 6 days after the final melarsomine injection from respiratory distress. The patient had been placed under some, but not strict, exercise restriction between the first and third melarsomine injection. The remaining four owners reported their pets’ death within 4 weeks of the final melarsomine injection, but no additional information was available. All four remaining patients had been placed under strict exercise restriction and confined to a small space between the first and third melarsomine injections.

### Therapy adherence

#### Medication

The average adherence rate for all prescribed medications exceeded 80%, with less than 3% of owners reporting non-administration of any prescribed medication throughout treatment (Table 6). According to owner reports, doxycycline was administered as prescribed in 97% (484/499) of patients. Owners reported consistent adherence to prednisone administration as prescribed in 95% (478/505) of patients after the first injection and 94% (349/370) for the subsequent second and third injections. Tramadol was reported to be administered as prescribed in 82% (410/503) of patients after the first injection and increased to 86% (318/369) for the second and third injections. In addition, owners reported that heartworm preventives were consistently administered in 89% (455/509) of patients after evaluation, 91% (473/518) after the first injection, 94% (387/412) after the second and third injections, and 92% (192/209) after post-injection microfilaria testing. Among patients whose owners completed the intake questions at all four relevant appointments, 131/175 (75%) were given heartworm preventives with no lapses in therapy, while 44/175 (25%) received medication, but experienced occasional lapses in therapy since evaluation.

**Table 6:** Owner-reported medication adherence

Medication	Time frame	Adherence	Number yes	Total number	Percentage (%)
Doxycycline					
	Prior to injection 1				
		Administered as prescribed	484	499	97
		Some not administered	15	499	3.0
		None administered	0	499	0
Prednisone					
	Following injection 1				
		Administered as prescribed	478	505	95
		Some not administered	22	505	4.4
		None administered	5	505	1.0
	Following injections 2/3				
		Administered as prescribed	349	370	94
		Some not administered	20	370	5.4
		None administered	1	370	0.3
Tramadol					
	Following injection 1				
		Administered as prescribed	410	503	82
		Some not administered	84	503	17
		None administered	9	503	1.8
	Following injections 2/3				
		Administered as prescribed	318	369	86
		Some not administered	42	369	11
		None administered	9	369	2.4
Heartworm preventive					
	Prior to injection 1				
		No lapses in therapy	455	509	89
		Some lapses in therapy	52	509	10.2
		Not administered	2	509	0.4
	Prior to injections 2/3				
		No lapses in therapy	473	518	91
		Some lapses in therapy	40	518	7.7
		Not administered	5	518	1.0
	Prior to post-injection microfilaria test				
		No lapses in therapy	387	412	94
		Some lapses in therapy	18	412	4.4
		Not administered	7	412	1.7
	Prior to post-injection antigen test				
		No lapses in therapy	192	209	92
		Some lapses in therapy	14	209	6.7
		Not administered	3	209	1.4

#### Exercise restriction

The average adherence rate for exercise restriction exceeded 75% throughout treatment (Table 7). Among patients whose owners completed the intake questions at both relevant appointments, 128/401 (32%) were placed under strict exercise restriction and confined to a small space for 60 days as prescribed, 212/401 (53%) were placed under some form of exercise restriction, and 61/401 (15%) were not placed under exercise restriction. Among owners, 175/385 (46%) and 138/285 (48%) reported restricting activity for 24 h a day after the first and second/third melarsomine injections, respectively. The median duration of exercise restriction reported was 23 h per day (IQR, 12) following each melarsomine injection.

**Table 7:** Owner reported location for exercise restriction following melarsomine injections

Intake response	Time frame	Number of dogs	Total dogs	Percentage (%)
Confined to a small space only				
	Between injection 1 and injections 2/3	230	520	44
	Between injections 2/3 and post-injection microfilaria test	193	414	47
Sometimes confined to a small space				
	Between injection 1 and injections 2/3	184	520	35
	Between injections 2/3 and post-injection microfilaria test	120	414	29
Free access to large space/No restriction				
	Between injection 1 and injections 2/3	106	520	20
	Between injections 2/3 and post-injection microfilaria test	101	414	24

## Discussion

### Financial barriers

Our results demonstrate that effective and safe heartworm disease treatment can be provided at a lower cost by using a technician-dependent, high-volume outpatient community medicine model. A low cost high-volume, outpatient care setting requires resource maximization, including financial resources as well as time and labor. When evaluating priorities based on restricted resources, it is essential that the quality of medicine provided to patients, and the owner’s experience, is not compromised. To strike this balance, we developed a modified treatment protocol that (1) incorporated short in-clinic post-injection observation periods and delegated continued patient monitoring to pet owners, eliminating the need for extended post-injection kenneling and monitoring at the clinic and enabling dogs undergoing heartworm treatment to be managed as outpatients; (2) minimized the use of costly ancillary diagnostic aids like radiographs and echocardiograms, as the results would not alter the treatment plan for the majority of patients; and (3) maximized the use of trained VT/VAs working under DVM supervision, allowing for safe and efficient treatment of up to 25 dogs per day. These measures, in combination, contribute to a reduction in the overall cost of treatment to owners.

#### Minimization of ancillary diagnostics

While AHS guidelines discuss radiography, echocardiography, and other laboratory tests as useful adjuncts for assessing an animal’s cardiopulmonary and overall health status, there is no specific AHS protocol for pre-melarsomine therapy diagnostic workup outside of patient history, physical examination, and antigen and microfilaria testing. The authors acknowledge that radiographs and echocardiograms can help identify the extent of cardiopulmonary disease associated with heartworm infection and thus may be helpful in evaluating the likelihood of post-melarsomine injection complications [5, 16]. However, echocardiography is not commonly available in high-volume community clinics. Radiography may be available but presents logistical challenges in a high-volume setting and may still be financially out of reach for some pet owners. Considering most patients in this study were class I or II, the results of these diagnostics would not have impacted their course of treatment. Instead, we elected to perform a blood chemistry panel in conjunction with a thorough history and physical exam as a feasible and cost-effective way to identify comorbidities that may complicate melarsomine therapy. This approach enables deferral of treatment until the condition can be stabilized at a full-service veterinary clinic or prescription of non-arsenical heartworm treatment as needed.

#### Minimization of in-clinic monitoring

While the AHS guidelines do not recommend hospitalization with close monitoring following melarsomine injections, AHS notes that hospitalization is generally recommended during treatment [17]. An informal review of veterinary message boards and veterinary hospital websites suggests that numerous practitioners opt to hospitalize patients for 12–36 h following melarsomine injections. By implementing a technician-dependent operational model, we could administer treatment and monitor patients for 2.5–4.5 h following each injection. This time window was designed to accommodate patient discharge procedures and aligns with the period during which most serious, immediate-type hypersensitivity reactions would be detected [18, 19]. By giving specific instructions regarding signs to watch for and how to contact a trained VT/VA and DVM after business hours, we were able to delegate continued patient monitoring to the pet owner.

#### Maximization of VT/VAs

As with many areas of practice, additional training coupled with the delegation of non-DVM tasks to VT/VA may also allow DVMs to provide additional or lower cost services for non-heartworm patients, maximizing their impact. The ability to delegate administration of melarsomine or other described tasks to VT/VAs may vary depending on individual state laws. DVMs should consult their state veterinary laws prior to delegating tasks associated with heartworm treatment to non-DVMs.

### Retention

Heartworm treatment according to AHS guidelines asks a lot of pet owners. It is a lengthy process with many steps, appointments, and medications to administer within the first 4 months (from evaluation to 4 weeks after the third melarsomine injection). While we attempted to mitigate financial barriers with our high-volume, outpatient heartworm treatment model, we recognize that owners experience multiple barriers to obtaining veterinary care and often experience them simultaneously [6]. It is also important to note that the study period overlapped with the height of the COVID-19 pandemic. During this time, owners may have encountered additional barriers to attending appointments and complying with treatment protocols, such as changes in financial situations, housing stability, access to transportation, work schedules, or owner health [20, 21]. Due to the prolonged period of treatment, and numerous barriers that exist with each step, we felt that it was important to share the retention rate at each stage of the heartworm treatment process, as it is not well documented in the literature.

We found that 97% of patients that began treatment completed all three melarsomine injections and 46% returned for antigen testing 9 months after the third melarsomine injection. Although 16% of patients were being fostered at the start of treatment, our findings do not show a significant difference when comparing ownership status. Despite the numerous obstacles noted above, these retention rates are excellent and comparable to that of patients seen in both tertiary referral centers or shelter settings [12, 22]. Our findings align with Maxwell et al., in which 36% of patients returned for post-treatment antigen testing [12]. Retention for post-treatment antigen testing was somewhat higher, but still relatively low, in another study, where documented antigen test results could only be obtained for 44/75 (59%) of dogs treated for heartworm in a shelter setting and then adopted [22].

Whereas published information for appointment no-shows in a veterinary setting is lacking, it is well-studied in human medicine. An appointment lead time of 8 weeks or longer significantly increases probability of no-shows [23]. During the data collection period for this study, appointments for all stages of heartworm treatment were scheduled at the initial evaluation appointment, so the lead time for most appointments was well beyond 8 weeks. All types of reminder systems, including telephone, text message, and email have been shown to improve appointment attendance and improve rescheduling of appointments that owners cannot keep [24]. Whilst we employed two measures of reminder notifications (telephone and email), additional measures tailored to the population served may be required to increase return rates.

Retention rates are often omitted or solely discussed as a problem to be resolved. We wanted to underscore one benefit of documenting and reporting treatment nonadherence resulting from both medical and non-medical factors. By distinguishing between patients that were excluded from treatment due to abnormalities detected in blood work or adverse reactions to a melarsomine injection, and those that did not return for other reasons, we were able to assess the rate at which patients were switched to a non-arsenical heartworm treatment or referred to a full-service veterinary clinic. These data help us understand the proportion of patients with severe concurrent medical conditions, or patients with the most severe clinical signs like caval syndrome, that were not resolved by our heartworm treatment model. We hope that by reporting these measures, we will encourage future research to assess factors that contribute to patient retention and develop additional methods of mitigating barriers to retention.

### Treatment success

Using our high-volume setting AHS protocol, 99% of dogs were NAD at post-treatment antigen testing 9 months after the third melarsomine injection. Our findings are consistent with first-line models previously reported in private practice or tertiary referral centers in which treatment success rates using the AHS protocol ranges from 88% NAD at 7 months after the third melarsomine injection (*n* = 50) [12] to 100% 6 months after the third melarsomine injection (*n* = 12, *n* = 15) [25, 26].

Owner-reported adherence with administration of heartworm preventives during heartworm treatment reported in this study was much higher than general heartworm preventive adherence reported in the literature. While monthly adherence to heartworm preventive medication has been estimated at 26–36% [27], the adherence observed during this study was more than twice as high. This heightened adherence may be expected in dogs with active and diagnosed heartworm disease, in contrast to healthy or undiagnosed dogs. Nonetheless, 25% of patients experienced some lapse in monthly preventive administration. To further increase adherence to heartworm preventives, we recommend the addition of a sustained-release injectable preventive option, which has been found to have twice the adherence of monthly preventive options [28].

### Adverse reactions

Our results indicate that the timing of the sedative administration relative to the melarsomine injection had a significant impact on the number of adverse reactions observed at the clinic. When owners administered the sedative prior to bringing the patient to the clinic, its effects began wearing off before the patient was discharged. In contrast, however, when VT/VAs administered the sedative after the patient’s arrival at the clinic, its effects were sustained for the duration of the patient’s appointment. While it was not a focus of this study, this suggests an optimal window for melarsomine injection post-sedation to maximize efficacy and minimize masking of observable adverse reactions.

Although our assessment of post-injection adverse reactions relied on observing kenneled pets in the clinic after receiving a mild sedative that morning, the sedative had limited practical impact on our ability to observe adverse reactions before discharging the patient to their owner. This is evident, as we still observed behavioral adverse reactions suggestive of pain and/or FAS in a high number of dogs. Consistent with the literature [12], adverse reactions observed by VT/VA were common, but major adverse reactions and death were rare.

Behavioral reactions were among the most observed adverse reactions, especially when the patient was at the veterinary clinic. These findings are unsurprising, considering that being physically in a clinic setting can contribute to increased manifestation of FAS [29–31]. While creating a low-stress environment and employing low-stress handling techniques can help mitigate these reactions, some level of occurrence is still expected. Although a sedative can potentially pose challenges to identifying adverse effects of the melarsomine injection due to the depressant effects, it can concurrently reduce FAS for the duration of an injection appointment. In high-volume clinic settings, additional measures are necessary to ensure efficient work by VT/VAs and proper melarsomine placement while minimizing patient injury during injection. Given the use of sedatives not only facilitates restraint by VT/VAs, reducing patient movement during injection, but also contributes to the reduction of patient FAS, we recommend the use of short-acting or reversible sedatives, in combination with adequate analgesia. This approach is preferable to heavy and long-acting sedatives that could mask adverse reactions observable in a clinic setting.

Post-injection pain was one of the most reported adverse reactions observed by both VT/VAs and owners. These findings are not surprising given the requirement for melarsomine, which is a highly irritating drug known to potentially cause substantial tissue damage [18, 19], to be administered in a region near spinal nerves, deep within the epaxial muscles. Moreover, this administration typically involves a considerably large volume of fluid, with up to 4 ml per injection site, and sometimes larger volumes divided between the two injection sites. Even though AHS does not make recommendations for pain medication to be administered following melarsomine injections, we believe that the administration of an analgesic is more humane and can help mitigate the post-injection pain response for the patient, as well as help the owner manage the patient’s comfort level at home. While our study was not designed to test the impact of analgesics on adverse reactions, the finding that VT/VAs observed fewer adverse reactions following the third melarsomine injection, a period at which we would expect a higher incidence of pain effect due to the short window between the second and third melarsomine injections, suggests that the tramadol administered following the second melarsomine injection helped mitigate these effects. This is further supported by a recent study by Ward et al. which found a low incidence of injection site reactions when a mu-agonist opioid was administered prior to melarsomine injections [22]. Given the low financial cost associated with analgesics and the benefits associated with their use, we support the continued administration of analgesics for patients undergoing heartworm treatment.

The authors would consider alterations to this protocol to further enhance pain management, including earlier administration to allow the analgesic to take effect before the melarsomine injection is administered, as well as the incorporation of multimodal pain management. At the time of protocol development, tramadol was selected for its safety and efficacy as an analgesic, its compatibility with other prescribed medications, and its ease of dispensing to clients for at-home administration, ensuring continued pain management for several days post-injection. Recent studies question the effectiveness of tramadol, necessitating a review of its inclusion in the protocol [32]. We recommend considering alternatives based on several factors, including the safety of the options when administered in combination with steroids, their effectiveness and duration, ease of administration for pet owners, potential for misuse by pet owners, and adherence to regulatory requirements.

Adverse respiratory reactions can develop or worsen during heartworm treatment, as dying worms cause pulmonary inflammation and pulmonary thromboembolism (PTE) [16]. According to Hirano et al., signs of PTE are most common within 7–10 days of melarsomine injection but can occur up to 4 weeks later. Adherence with two components of the treatment protocol play an important role in the occurrence of pulmonary inflammation and PTE during heartworm treatment: doxycycline [33] and exercise restriction [5, 34]. While AHS guidelines recommend implementing exercise restriction at the time of diagnosis and continuing through 6–8 weeks following the final melarsomine injection [5], in practice, strict exercise restriction over an extended period is extremely difficult for many dog owners to implement. Factors such as lack of equipment or space for confining the pet, the pet’s temperament, or other household members can all interfere with implementing long-term exercise restriction. With these challenges in mind, owners were advised to restrict activity for a more manageable 60 days total. Consequently, we collected owner-observed adverse reactions within the first 10 days of melarsomine injection as well as adherence with doxycycline and activity restriction protocols throughout treatment.

Our findings, indicating that 19% of owners reported respiratory reactions within 10 days of a melarsomine injection, are consistent with the literature, which shows a wide range of reported rates (2–48%) depending on the methods of monitoring, classification, and reporting of respiratory signs [12, 22, 35–37]. Given that all patients completed doxycycline prior to their first melarsomine injection, and adherence with exercise restriction was greater than 75% after each melarsomine injection, it is unsurprising that we also observed a low mortality rate. Within the 4-week window in which PTE is most likely to occur, 1.3% of patients that began treatment died. Mortality rates for heartworm treatment protocols following AHS guidelines range from 0 to 4% [25, 26, 35], with one study reporting a particularly high mortality rate of 14%, primarily among class II or caval syndrome patients [12]. While two of the seven deaths occurred within the 10 days of a melarsomine injection, an additional four deaths occurred between the 10-day and 4-week window. Unless a conversation was initiated by the owner, organizational policy prohibited detailed inquiry into a patient’s death that occurred outside of the clinic if more than 2 weeks had passed since the date of death. Consequently, some information may be missing regarding the details of some patients’ deaths. Therefore, we recommend that additional studies extend the observation window to encompass the full 4-week period during which PTE can occur.

## Limitations and future directions

92% of patients in our study had class I heartworm disease, with only a single class III patient. We believe this may be largely attributed to the early diagnosis of heartworm infection by screening patients during high-volume healthy pet wellness visits. It is important to note that while our heartworm treatment model is not limited to class I or II cases, the healthy pet wellness model used at the study sites does not accommodate patients with signs of disease or comorbidities. This can further bias the treated cases towards class I heartworm disease, which is often asymptomatic. While this might impact the applicability of our data to populations with a higher percentage of class III patients, the effectiveness and safety of this model for dogs with severe heartworm disease is limited. Such cases often necessitate additional diagnostics, extended in-hospital treatment, and intensive monitoring, making them unsuitable for the high-volume heartworm treatment approach evaluated here [38].

Many studies are retrospective in nature and often exclude patients with incomplete treatment or follow-up testing from their analysis. In our case, the primary aim of this study was to assess the safety and efficacy of a specific treatment protocol and process flow for individual animals. Consequently, we similarly chose to exclude patients with incomplete treatment and follow-up testing from our analysis of treatment success. While our retention rate at post-injection antigen testing was comparable with what is typically reported in the literature [12], it is important to note that we do not have outcome data for over half of the patients that completed treatment. Considering the potential of treatment-non-adherent patients to remain infectious and therefore act as reservoirs for heartworm disease, we recommend that researchers interested in assessing the population-level impact of heartworm treatment include the number of patients that start but fail to complete heartworm treatment in the calculation of treatment success.

Data collected for this study were part of standard clinical care at each of the study clinics. As a result, therapy adherence and owner-observed adverse reactions were reported using self-administered questionnaires. It is thus important to acknowledge one limitation of our study, which is the potential for information bias (or self-reporting bias). If owners underreported responses due to social desirability bias, our results might overestimate adherence rates. On the other hand, if owners reported inaccuracies due to difficulty in recalling past events (recall bias), our results could either underreport or overreport adverse reactions, depending on whether errors stemmed from an inability to remember which adverse reactions occurred at all or which occurred within the 10-day observation period. The influence of self-reporting is widely recognized in the literature and strategies to help reduce the impact are well established [39]. When surveying owners about therapy adherence, we sought to reduce social desirability bias by basing our self-administered questionnaires on previously published materials [12] and cross-checking owner responses with the medical record where applicable. To mitigate recall bias, owners received an information packet listing the adverse reactions they needed to report. However, because we surveyed owners at the subsequent visit, the questionnaires were administered approximately 3–4 weeks after the events in question. As self-administered questionnaires are commonly used in veterinary medicine, we encourage practitioners to internally validate their measurement tools to ensure owners are comfortable and confident in their reporting, and to ensure patient history is accurate.

## Conclusions

As the prevalence of heartworm disease continues to grow, there is a critical need to offer more affordable heartworm treatment. At present, melarsomine-based heartworm treatment is an expensive, life-saving treatment that is rarely offered at a low cost in high-volume community clinic settings. Our study is the largest known prospective clinical study based on AHS guidelines to date, and it is the first to evaluate heartworm treatment in a high-volume outpatient clinic setting. Our findings demonstrate that implementing a technician-leveraged approach to heartworm treatment allows for effective and safe melarsomine therapy in a high-volume outpatient setting and requires only minor modifications to AHS guidelines. With the protocol employed in this study, veterinary care providers can minimize the cost required to treat heartworm disease and increase access to high quality heartworm treatment by reducing the financial barriers to obtaining care.

## Data Availability

The de-identified datasets used and/or analyzed during the current study are available from Emancipet upon reasonable request. Data requests can be made by email to: research@emancipet.org.

## Abbreviations

AHS: American Heartworm Society
ALKP: Alkaline phosphatase
ALT: Alanine aminotransferase
BID: Twice per day
BUN: Blood urea nitrogen
DVM: Veterinarian
FAS: Fear, anxiety, and stress
GLMM: Generalized linear mixed model
IM: Intramuscular injection
NAD: No antigen detected
PO: Orally
SID: Once per day
SNAP: IDEXX SNAP Heartworm RT Test
VT/VA: Veterinary technician/veterinary assistant
WITNESS: Zoetis WITNESS Heartworm Rapid Test
